# Effectiveness of Administering a Mixture of Lactic Acid Bacteria to Control *Salmonella* ser. Enteritidis Infections in Broilers

**DOI:** 10.3390/ani12030374

**Published:** 2022-02-03

**Authors:** Yu-Jin Kim, Sungsu Youk, Chang-Seon Song

**Affiliations:** 1 College of Veterinary Medicine, Konkuk University, Neungdong-ro, Gwangjin-gu, Seoul 05029, Korea; yujinml@hanmail.net; 2 Exotic and Emerging Avian Diseases, Southeast Poultry Research Laboratory, National Poultry Research Center, Agricultural Research Service, United States Department of Agriculture, Athens, GA 30605, USA; sungsu.youk@usda.gov; 3 KCAV Co., Ltd., Neungdong-ro, Gwangjin-gu, Seoul 05029, Korea

**Keywords:** *Salmonella* ser. Enteritidis, probiotics, short-term administration, SE detection

## Abstract

**Simple Summary:**

*Salmonella* infection is one of the main causes of food poisoning through poultry consumption. Among the various methods used to control this infection, the use of lactic acid bacteria is economical, with little risk of developing antibiotic-resistant bacteria. We selected three *Lactobacillus* spp. capable of inhibiting *Salmonella* proliferation in vitro and administered their mixture to 1-day-old chicks to investigate their effect. We suggest that the *Lactobacillus* mixture formulated in this study aids in protecting poultry farms from *Salmonella* contamination, further securing food safety.

**Abstract:**

Non-typhoidal *Salmonella* spp. cause persistent asymptomatic infections in poultry. The consumption of *Salmonella*-infected poultry products is associated with food poisoning. One of the pathogens that causes such infections is *Salmonella* ser. Enteritidis (SE). Therefore, alternative measures are required for better control of salmonellosis and to reduce potential antibiotic use. Here, the efficacy of a mixture of lactic acid bacteria (LAB), formulated based on competitive exclusion, was evaluated. The LAB mixture was administered to 1- to 20-day-old chickens using different schemes; the chickens were then inoculated with an SE strain, which was previously identified to be prevalent in broiler breeder farms. Even with short-term administration, the group treated with LAB exhibited lower SE isolation levels in the spleen and cecal content and greater weight gain than that in the control group. This protective efficacy of LAB was retained even after two weeks without LAB administration. According to the results of animal experiments and field tests, evidence of SE infection was absent after treatment of the animals with the LAB formulation used in this study. Thus, this LAB mixture can be used as a potential strategy for protecting poultry farms from *Salmonella* contamination. This will also help reduce potential antibiotic use.

## 1. Introduction

*Salmonella enterica* infections, specifically human salmonellosis, caused by non-typhoidal *Salmonella*, are a major public health concern worldwide. The consumption of contaminated poultry products is primarily responsible for human salmonellosis [1]. Among the numerous *Salmonella* serovars, *S. enterica* ser. Enteritidis (SE) is the most predominant [2]. SE causes food poisoning in humans and is responsible for economic losses to the poultry industry, primarily due to marked growth depression [3]. This pathogen spreads across poultry farms through vertical transmission from infected hens or horizontal transmission among young chicks. It causes persistent infections and may be a predisposing factor for other pathogenic infections. Many strategies have been used to control *Salmonella* contamination, including vaccination of chickens and the administration of antimicrobial agents [3]. Vaccination results in additional economic burden and requires intensive labor, whereas the use of antimicrobial agents may result in the emergence of resistant strains. Therefore, alternative measures using different biological products as feed additives have been developed for better control of salmonellosis [4].

Lactic acid bacteria (LAB) have certain benefits; they can be obtained from natural sources; are generally non-toxic to humans, animals, and commensals, and are relatively inexpensive, unlike many antibiotics [5]. These characteristics have rendered the use of LAB an attractive alternative for controlling SE infections in commercially important animals [6]. In addition, probiotics, including LAB, are conventionally used as feed additives to promote animal growth and health [5]. Effectively selected LAB have contributed to the establishment of robust health and prevention of *Salmonella* infections in poultry in an experimental setup [7,8]. In this study, we selected LAB capable of suppressing the SE strain isolated from a commercial farm, evaluated the efficacy of different LAB administration methods in the laboratory, and applied the most biologically effective and economically efficient methods identified in laboratory experiments to actual farm settings for checking the effectiveness of the selected LAB as an adequate SE control method.

## 2. Materials and Methods

### 2.1. Bacterial Strain Preparation

LAB were cultured in Difco De Man, Rogosa and Sharpe (MRS) broth (BD, NJ, USA) for 48 h at 37 °C. We selected three strains of LAB (LAB 15-64, *Lactobacillus vaginalis*; LAB 15-68, *L. plantarum*; and M3, *L. helveticus*) based on the results of an agar overlay test that was used to assess 128 LAB samples obtained from Korean fermented foods and healthy birds. The agar overlay test was used to verify competitive exclusion and the production of antibacterial substances, such as bacteriocins, by the LAB using an in vitro selection method. The three selected strains were cultured separately in 35 mL of the MRS broth. Cells were centrifuged at 1400× *g* and 4 °C for 20 min, and the MRS supernatant was discarded. Pelleted LAB were resuspended in 45 mL of autoclaved distilled water; centrifugation and resuspension were repeated thrice. After the final wash, pelleted LAB were resuspended in 0.5% skimmed milk, mixed to prepare a LAB solution with the same number of single cells of each strain, and lyophilized. The lyophilized LAB pellets were resuspended in distilled or tap water, and a suspension containing 10^8^ colony-forming units (CFUs) of bacteria was administered to each animal.

An SE strain (F17-362, provided by Konkuk University, Seoul, South Korea), isolated during environmental surveillance at a farm, was used as the experimental strain for both in vitro and in vivo tests. Bacterial stocks were incubated in Bacto Tryptic Soy (TS) broth (BD) at 37 °C for 18 h. For the challenge test, they were washed with autoclaved distilled water and resuspended at a concentration of 10^10^ CFU/mL.

### 2.2. Agar Overlay Test

The agar overlay test was performed by modifying a previously established method [9]. We prepared two MRS agar plates at different concentrations. One had a base layer containing 1.5% agarose (Difco MRS broth, BD; Micro agar, Duchefa Biochemie, Haarlem, The Netherlands). This MRS plate had three small, equally spaced holes for 45–50 µL of LAB filler agar. We used 0.8% MRS agar and 0.7% TS agar (Bacto TS broth, BD; Micro agar, Duchefa Biochemie) to prepare the filler and topper layers, respectively. The agar was melted and cooled to 50 °C prior to use. We mixed up to 10^7^ CFU of LAB and *Salmonella* into each agar solution before pouring it onto a solid base layer. After the top layer solidified, the plates were incubated at 37 °C for 24 h, and the diameters of the *Salmonella* inhibition zones were determined. The measured values (mm) were divided by the log value of the concentration of LAB used for log_10_ CFU. The unit was expressed as the ratio of the inhibitory zone area per unit concentration (anti-*Salmonella* ability of specific LAB = value from agar overlay test result/concentration of LAB used in the experiment).

### 2.3. LAB Application and SE Challenge in Specific Pathogen-Free (SPF) Chickens

All animal procedures were approved by the Institutional Animal Care and Use Committee of Konkuk University (KU20159). One-day-old SPF chickens (*n* = 106; Namduck, Korea) were divided randomly into three different LAB treatment groups. Analysis of variance (ANOVA) showed no significant differences in the average body weight among the groups on the day of commencement of LAB treatment. Each chick in the groups treated with LAB was orally administered 1 mL of 10^8^ CFU of the LAB mixture once daily using the following schedule: Group 1 and 2 (G1 and G2) were administered the LAB mixture for 3 d and Group 3 (G3) was administered the LAB mixture for 20 d. For comparison, one control (no treatment) group was used with G1, whereas a common control group was used for G2 and G3, since these two groups were challenged with SE on the same day. Animals in the control groups were housed separately before the pathogen challenge. After completion of the treatment schedule, the birds were orally challenged with 1 mL of 10^10^ CFU of SE at the age of 4 d (G1 and control) or 21 d (G2, G3, and control). The different groups of animals were raised in individual isolators where temperature and humidity were maintained, and they were provided ad libitum access to water and feed.

Changes in weight and clinical symptoms (e.g., anorexia, diarrhea, and moribundity) were observed daily until the day of SE re-isolation from each group. Up to 10 chicks from each group were euthanized on 1, 3, and 17 d (only part of G1) post challenge (dpc) for *Salmonella* re-isolation. Cecal tonsil contents, livers, and spleens were weighed, homogenized, and cultured in buffered peptone water (BPW) broth (BD) at 36 °C for 18 h and in Rappaport-Vassiliadis (RV) broth (Oxoid, Hampshire, UK) at 42 °C for 36 h. The BPW supernatants were used for *Salmonella* spp. detection using polymerase chain reaction (PCR) with specific primers (Forward: 5′–AATATCGCTTCGTACCAC–3′, Reverse: 5′–GTAGGTAAACGAGGAGCAG–3′, 274 bp). The supernatants from the RV broth were spread over *Salmonella* ChromoSelect Agar, Improved (Sigma-Aldrich, Burlington, MA, USA).

### 2.4. LAB Application in Farm Settings

For field experiments, we selected two broiler farms from a list of farms that violated relevant laws and regulations at least two consecutive times during routine inspections of slaughterhouses for SE contamination. Farm 1 consisted of four buildings with 10,000 chicks each. Farm 2 consisted of eight buildings with 10,000 chicks each. Lyophilized LAB pellets were provided to each farm and were supplemented with water and feed for 3 d. The LAB pellets were dissolved directly in drinking water in a water tank. The estimated LAB consumption via water was 10^8^ CFU per bird per day. LAB pellets were also supplemented with feed, and the chicks were allowed ad libitum access to feed. We re-isolated SE and other *Salmonella* spp. from the environment based on a previously published method and schedule for environmental *Salmonella* detection [10] as follows: before animal entry (after the cleaning routine), after animal entry, during raising of the animals (days 10–12), and before moving the animal to the slaughterhouse. Re-isolation from the animals was performed using the same method described above: after animal entry (day 1), after probiotic administration (days 4–5), and after changing the feed (days 10–12). Tested animals were selected by farm workers during routine animal check-ups. An average of 1% of the animals in a building were tested for *Salmonella* infection. These animals were moved to the laboratory, either dead or alive, according to the farm conditions. The final re-isolation process was performed during routine SE inspection of the slaughterhouse.

### 2.5. Statistical Analyses

All data were analyzed using GraphPad Prism version 8 software for Windows (GraphPad Software, San Diego, CA, USA). ANOVA, Student’s *t*-test, and Fisher’s exact test were used to determine significant differences among the groups for the initial weight, weight gain due to experimental conditions, and re-isolation rate, respectively. Chi-square test was performed only for some re-isolation samples. Statistical significance was set at *p* < 0.05.

## 3. Results

### 3.1. In Vitro LAB Selection Test

We assessed 128 strains of LAB to determine the levels of competitive suppression of SE using the agar overlay test. We sorted LAB in the order of high anti-*Salmonella* capacity per unit concentration using this test (Table 1). Approximately 10% of the tested probiotics showed anti-*Salmonella* capacities above 1.6. The results also suggested that *Lactobacillus* spp. were generally more effective than other species at inhibiting *Salmonella* growth. Among the top six LAB, three different species of LAB were selected, based on the growth rate (LAB 15–64, *L. vaginalis*; LAB 15–68, *L. plantarum*, and M3, *L.* *helveticus*).

### 3.2. Laboratory Pathogen Challenge

**Figure 1:**
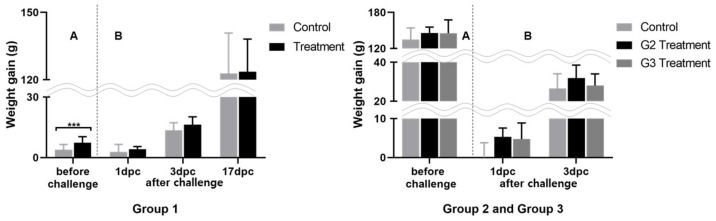
Weight gain differences post LAB administration before and after SE challenge, *** *p* < 0.001.

We treated animals for SE infection with a mixture of selected LAB under different administration schedules. The animals were grouped, and the experimental time points were selected based on the experimental design and the time required for the formation of chicken commensals [11]. With this approach, we aimed to determine the most efficient LAB administration program. The clinical response to this treatment was determined by observing the changes in morbidity, mortality, and weight. To determine the efficacy of LAB against SE, based on a preliminary study, we orally inoculated a high dose of SE (1 mL of 10^10^ CFU), enough to cause mortality by SE infection. Based on the results of our preliminary examination (three deaths in 20 one-day-old chickens, data not shown), we expected to determine the efficacy of the selected LAB by observing the mortality caused by the high-dose challenge; however, no mortality was observed in this study. The average weight gain in the experimental and control groups before the SE challenge was insignificant (Figure 1), although weight gain variance was larger in the control groups than that in the groups administered LAB by the end of the experiment. After the SE challenge, animals in the groups administered LAB gained more weight with a little variance. This tendency was more pronounced immediately after the SE challenge as the animals were young.

SE is an opportunistic pathogen, and infection can recur in the body under stressful conditions. We hypothesized that SE would be in a latent stage in the immune-related organs of an animal, such as the spleen and liver [12]. We measured the proportion of SE involved in latency and that present in the intestine, which can immediately cause clinical symptoms (Table 2). Although no differences in the proportion of SE following re-isolation from the organs were observed at 1 dpc in G2 and G3, it was significantly lower in every experimental group, particularly in the spleen, at 3 dpc than that in the control group (*p* < 0.05). Cecal content was analyzed using PCR and re-isolation to assess the presence of SE at 1, 3, and 17 dpc. In the groups administered LAB, SE was detected in the cecal content in proportions similar to that in the control groups but was absent or present in very small proportions in the evaluated organs.

Panels A and B correspond to weight gain difference between the initial and before challenge, and weight gain difference after challenge respectively; *** *p* < 0.001, compared to that of the control group at different time intervals, by Student’s *t*-test.

### 3.3. Field Tests

A field test was conducted at two farms using a selected short-term administration schedule (Table 3). *Salmonella* spp. were not detected in the environmental samples from the group administered LAB in Farm 1, whereas the control group in the same farm was infected with *Salmonella* spp. In Farm 2, *Salmonella* ser. Montevideo was detected in the environmental samples from both birds administered LAB and control birds. SE was not detected in any of the animal samples from Farm 1. Conversely, *Salmonella* spp. infection was detected in both the groups of Farm 2 when testing was performed immediately after the introduction of newly hatched chicks. However, *Salmonella* spp. were absent in the group administered LAB until the end of the sampling period, whereas the control group birds were contaminated with *Salmonella* spp. during the feed change and slaughterhouse stages.

## 4. Discussion

LAB are primarily used as feed supplements during animal breeding to promote weight gain. Improved productivity after the administration of probiotics, such as *L. sakei* or *Bacillus subtilis*, is related to the generation of a favorable microenvironment by these probiotics, specifically facilitating an increase in the villus height and crypt depth of the intestinal epithelium and the establishment of a low-pH intestinal environment, which is helpful for nutrient absorption [13,14,15].

The recent ban on the use of antibiotics for growth promotion and disease prevention in many regions has led to the use of LAB as an alternative. There are no distinct clinical symptoms associated with SE when it is the only pathogen on the farm; thus, it remains undetected by farm workers. This makes it difficult to find and eradicate SE in farms, which may lead to the generation of asymptomatic carriers in fully grown animals. Here, we demonstrated that LAB that were selected based on competitive exclusion and production of antibacterial compounds of SE helped reduce SE re-isolation in young chicks.

*Salmonella* can enter the intestinal epithelial cells to form phagosomes via numerous host–pathogen interactions [16]. Young animals are vulnerable to pathogen invasion prior to the formation of robust physical, chemical, and biological barriers on the intestinal wall. Probiotics have been shown to reduce the harmful effects of SE through several approaches. One of these is the preoccupation of the SE adhesion site in epithelial cells. Other defense mechanisms of probiotics include stimulation of immunoglobulin A secretion, modulation of intestinal permeability, and production of inhibitory metabolites [17].

The host-adapted strain of SE can become an opportunistic pathogen, causing persistent systemic infections when multiple intestinal immune barriers stop functioning [18]. In this regard, 1-day-old chicks are considered more vulnerable to SE infection due to immature intestinal integrity. Thus, we started the administration of the LAB mixture to the chicks on day 1; SE infections decreased within 3 d of LAB administration. Additional advantages associated with LAB administration during this period are that it can help prevent SE invasion and subsequent systemic spreading, recurrence, and vertical transmission by competitive exclusion and organic acid or antimicrobial substance production [19].

SE reduction was observed not only in the long-term administration group (G3) but also in the short-term administration group (G2). In G2, SE challenge proceeded two weeks after stopping the administration of the LAB mixture; however, SE re-isolation in the organs and cecal content of G2 was found to be similar to that of G3. This further corroborates the information that the creation of an intestinal microenvironment is important for defense against SE. The sturdy intestinal tissues of chicks produced at the initial stage of LAB administration are also associated with positive effects, including effective absorption of nutrients, increased initial weight gain, and maintenance of body weight uniformity in the entire flock compared with that in the non-treated flock [20,21]. Therefore, if suitable probiotics are selected based on appropriate standards and methods and animals are treated at an early age, long-term administration may not be essential for creating sturdy intestinal conditions in commercial poultry. Compared to the long-term administration of LAB, this early initiation and adequate and short-term administration of LAB is a highly economical and efficient strategy for better management of poultry.

In the present study, we established an experimental model to distinguish between SPF chickens with and without SE infection. We used a notably high concentration of SE, which was frequently isolated during routine environmental inspections at a poultry farm. A previous study indicated that severe SE infections can cause mortality in young animals, depending on the strain [3]. The SE isolate used in this study caused no mortality or clinical symptoms, despite the exceptionally high dose of the challenge. Therefore, to improve the detection of SE contamination, re-isolation from immune organs and cecal contents along with clinical observation is strongly encouraged.

According to the results of the animal experiments and field tests, evidence of SE infection was absent after the treatment of animals with LAB. In a field test, *Salmonella* was coincidentally observed in the environment, and newly introduced chicks were already contaminated with *Salmonella* spp. No detection of *Salmonella* after LAB administration showed potential benefit of its use in a farm environment. The *Lactobacillus* mixture formulated in this study can be used as a potential tool for protecting the poultry farm from *Salmonella* contamination. This will also help reduce the potential use of antibiotics. 

However, a limitation of the present study is that weight variations in the long-term administered group (G3) were similar or even more than those in the control group, which indicates that long-term administration causes growth to either improve or deteriorate depending on the initial condition. Further experimental studies are required to determine the cause of this phenomenon.

## 5. Conclusions

In summary, this research describes an appropriate program for the administration of LAB. Our study showed that the prohibition of SE infection by meticulously selected *Lactobacillus* is effective when LAB have been administered at the right time, even if it has been administered for a short duration. By demonstrating the efficacy of LAB in controlling SE infection, this study provides an ideal approach to further study the prevention of infection by other animal-derived pathogens.

## Figures and Tables

**Table 1:** In vitro inhibitory effect of LAB against SE.

Range ^a^	Numbers ^b^	Species ^c^
2.0–1.8	5	*Lactobacillus* spp. (5) - LAB 15–64, *L. vaginalis*, measured 2.00; LAB 15–68, *L. plantarum*, 1.81
1.8–1.6	8	*Lactobacillus* spp. (8) - M3, *L. helveticus*, 1.79
1.6–1.4	11	*Lactobacillus* spp. (4), *Pediococcus* spp. (3), *Enterococcus* spp. (3), *Bacillus* spp. (1)
1.4–1.2	32	*Lactobacillus* spp. (17), *Bacillus* spp. (7), *Pediococcus* spp. (2), *Enterococcus* spp. (2), *Streptococcus* spp. (2), *Weissella* spp. (1), *Sporolactobacillus* spp. (1)
1.2–1.0	42	*Lactobacillus* spp. (29), *Bacillus* spp. (6), *Enterococcus* spp. (2), *Sporolactobacillus* spp. (2), *Weissella* spp. (2), *Streptococcus* spp. (1)
less than 1.0	29	*Bacillus* spp. (14), *Lactobacillus* spp. (9), *Enterococcus* spp. (3), *Pediococcus* spp. (1), *Sporolactobacillus* spp. (1), *Leuconostoc* spp. (1)
	128	

^a^ anti-*Salmonella* ability of specific LAB = value from agar overlay test result/concentration of LAB used in the experiment; ^b^ Number of LAB species used; ^c^ Number of species in parentheses. Three LAB strains selected for the animal studies are appended with the diameter measured.

**Table 2:** Different schedules of LAB mixture administration and SE re-isolation in chickens.

		LAB Administration for 3 d				LAB Administration for 20 d	
Re-Isolation		G1		G2		G3	
		Control	Treatment	Control	Treatment	Control	Treatment
1 dpc ^a^	liver	3/10 ^c^	1/10	0/8	0/7	0/8	0/8
	spleen	3/10	3/10	0/8	0/7	0/8	0/8
	cecal content	10/10	8/10	4/8	4/7	4/8	6/8
3 dpc	liver	1/10	1/10	0/8	0/8	0/8	0/8
	spleen	6/10	1/10 *	4/8	0/8 *	4/8	0/8 *
	cecal content	10/10	10/10	5/8	4/8	5/8	2/8
17 dpc	liver	2/10	0/10	na ^b^	na	na	na
	spleen	2/10	1/10	na	na	na	na
	cecal content	4/10	0/10 *	na	na	na	na

^a^ dpc, days post challenge; ^b^ na, not available; ^c^ no. of positive results/no. of examined specimens; * *p* < 0.05, compared to the control group, by Chi-square test or Fisher’s exact test

**Table 3:** Application of the LAB mixture in an actual farm setting and SE isolation from the farm environment and chickens.

Farm #	Group	Time of SE Isolation								
		Before Stocking	1-Day-Old Chickens (Time of Entry)		10-Day-Old Chickens		17–20-Day-Old (After Changing the Feed) ^c^		28–30-Day-Old Chickens (Before Moving to Slaughterhouse)	
		Environment	Environment	Chickens	Environment	Chickens	Environment	Chickens	Environment	Chickens
Farm 1	LM-treated ^a^	-	-	-	-	-	-	-	-	-
	Non-treated	-	-	-	+ (Sal spp.) ^b^	-	+ (Sal spp.) ^b^	-	-	-
Farm 2	LM-treated ^a^	-	-	+ (Sal spp.)	-	-	-	-	+ (S. Montevideo)	-
	Non-treated	-	+ (S. Montevideo)	+ (Sal spp.)	-	-	-	+ (S. Montevideo)	+ (S. Montevideo)	+ (Sal spp.)

^a^ LAB mixture-treated: same LAB mixture used in the laboratory experiment; ^b^ salmonella was confirmed using a colony polymerase chain reaction, but serovar details have not yet been identified; ^c^ size of the feed crumble is enlarged in this period as the feed was changed from that meant for chicks to that for broilers.

## Data Availability

The data presented in this study are available on request from the corresponding author.
